# Heart rate variability responses to personalized and non-personalized affective videos. A study on healthy subjects and patients with disorders of consciousness

**DOI:** 10.3389/fpsyg.2025.1560496

**Published:** 2025-04-03

**Authors:** Sandra Goizueta, Anny Maza, Ana Sierra, María Dolores Navarro, Enrique Noé, Joan Ferri, Roberto Llorens

**Affiliations:** ^1^Neurorehabilitation and Brain Research Group, Institute for Human-Centered Technology Research, Universitat Politècnica de València, València, Spain; ^2^IRENEA, Instituto de Rehabilitación Neurológica, Fundación Hospitales Vithas, València, Spain

**Keywords:** disorders of consciousness, minimally conscious state, unresponsive wakefulness syndrome, diagnosis, covert cognition, emotion recognition, heart rate variability

## Abstract

**Introduction:**

The diagnosis of patients with disorders of consciousness (DOC), including those in a minimally conscious state (MCS) and those with unresponsive wakefulness syndrome (UWS), remains a significant clinical challenge. Neurobehavioral assessment primarily relies on motor responses to commands, which are often difficult to interpret due to impaired comprehension and cognitive-motor dissociation, resulting in a high rate of misdiagnosis. While electrical, hemodynamic, and metabolic brain responses, combined with personalized stimuli, have shown promise in improving diagnosis, the role of cardiac activity—less intrusive and time-efficient—remains underexplored.

**Methods:**

This study investigated heart rate variability (HRV) responses to personalized videos of acquaintances versus non-personalized videos of strangers. The study included 17 healthy subjects and 11 patients with DOC. Cardiac responses were recorded and analyzed to compare responses to different stimuli and to examine differences between the two groups.

**Results:**

Healthy subjects exhibited significant differences in several HRV measures in response to both personalized and non-personalized stimuli, whereas patients with DOC did not demonstrate similar differences. Additionally, significant differences were observed in HRV measures between healthy subjects and patients with DOC.

**Conclusion:**

These findings suggest impaired emotional processing in patients with DOC. Further exploration of these differences may enhance diagnostic approaches for this patient population, particularly through the integration of HRV-based measures.

## 1 Introduction

Up to 30 to 40% of cases of severe acquired brain injury can lead to disorders of consciousness (DOC) (Giacino et al., 1991). This severely impaired clinical condition includes heterogeneous but defined clinical states, such as the unresponsive wakefulness syndrome (UWS), formerly coined as vegetative state, characterized by the presence of reflex motor responses, and the minimally conscious state (MCS), where some awareness of self or the environment is discernible (Giacino et al., 2002). The diagnosis of patients with DOC primarily relies on the presence of motor responses to different stimuli (Giacino et al., 2004). Paradoxically, the clinical assessment of the motor responses in this population, notably in relation to non-reflexive behaviors, represents an enormous challenge, since, by definition, the interaction of these patients with the environment is extremely limited (or inexistent) and they might suffer from impaired language comprehension (Giacino et al., 1991). In addition, the behavioral signs of consciousness can be fluctuating (Giacino et al., 2009) and be differently interpreted by the examiners (Schnakers et al., 2009; Van Erp et al., 2015; Wade, 2018). Furthermore, although some patients may have preserved cognitive functions, they may not be evident in motor responses due to a cognitive-motor dissociation (Giacino et al., 1991; Kondziella et al., 2016), resulting in a phenomenon of covert cognition (Schnakers et al., 2022). In consequence, the assessment of patients with DOC can have limited reliability and accuracy (Comanducci et al., 2020), and lead to a high rate of misdiagnosis (Gill-Thwaites, 2006) even using the Coma Recovery Scale–Revised (CRS-R) (Giacino et al., 2004), the most recommended instrument (Giacino et al., 2018; Kondziella et al., 2020; Scolding et al., 2021). For this reason, the most updated guidelines also suggest complementing the clinical assessment with multimodal evaluations (Stender et al., 2014).

Several neuroimaging methods, including positron emission tomography, functional magnetic resonance imaging and electroencephalography, are being increasingly investigated to minimize diagnostic errors and forecast the recovery of consciousness through resting-state or sensory-stimulation approaches (Alnagger et al., 2023; Jain and Ramakrishnan, 2020). Other physiological indicators, including electrodermal activity and electrocardiography, have also been employed for this objective, albeit with lower frequency (Alnagger et al., 2023; Liuzzi et al., 2023).

Heart Rate Variability (HRV), a measure of the variation in time between consecutive heartbeats, has been suggested as a potential physiological marker of emotional responses (Ahmad and Khan, 2022). Specifically, the HRV reflects the dynamic interplay between the sympathetic and parasympathetic branches of the autonomic nervous system (Ernst, 2017), which has been shown to be influenced by emotional experiences, even in the absence of verbal communication (Appelhans and Luecken, 2006; Buchanan and Tranel, 2009). This feature makes HRV a particularly interesting physiological phenomenon for investigating the awareness of patients with DOC. However, the number of studies examining the potential of HRV responses to detect signs of consciousness in this clinical condition is limited.

Most of these studies have explored how non-personalized music affects the emotional responses of patients with DOC (Riganello et al., 2019). Although some interventions have observed recognizable HRV responses to the music stimuli, the significant methodological limitations and variations among these studies hinder drawing reliable and consistent conclusions (Grimm and Kreutz, 2018). A preliminary study examined the HRV responses of 9 patients with UWS and 16 healthy subjects when exposed to different classical music pieces. The study found that regardless of the authors of the pieces and the quality (positive or negative) of the emotional responses, both groups showed differences from baseline in their power spectrum. Furthermore, the intensity (low or high) of the normalized low-frequency component (*nuLF*) values was correlated with the quality of emotional responses (Riganello et al., 2010). A subsequent case study on the effects of exposure to music in a patient with UWS reported an increase in the standard deviation of normal-to-normal intervals (*SDNN*) and the root mean square of successive differences (*RMSSD*) after two weeks of stimulation (Lee et al., 2011). This effect, however, was not confirmed by a later study on the impact of music therapy, which examined various neurophysiological measures, including HRV, specifically focusing on *RMSSD* (O’Kelly and Magee, 2013). The findings of this study were inconclusive and appeared to vary depending on the patient. Another study reported a significant reduction in the entropy of the HRV in a group of 9 patients with UWS when exposed to four musical pieces with varying structural complexity (Riganello et al., 2015). However, no noticeable differences were detected in healthy controls exposed to the same musical pieces under similar experimental conditions.

Only a few studies have employed personalized emotional stimuli, both of which utilized familiar voices, to examine HRV responses. A seminal study compared the responses of 12 patients with UWS when they were approached by a family member or by an unknown person who tried to replicate the same conversation, and found that among other HRV features, the *nuLF* was able to discern between both conditions with an accuracy of 69% (Dolce et al., 2008). A later study, involving 3 patients with UWS and one patient in MCS, explored reactions to either non-controlled affective conversations with their mothers or an unknown female voice reading a technical book and reported a consistent pattern of changes in three of the patients, including decreased heart rate, increased HRV, decreased power in the low frequencies, and increased power in the high frequencies (Gutiérrez et al., 2010).

Although the existing literature exhibits certain sensitivity of the HRV to emotional responses of patients with DOC, they have common limitations that hinder determining the reliability of HRV measures for investigating the level of consciousness. Firstly, most studies have only included a very limited number of patients with DOC (Dolce et al., 2008; Gutiérrez et al., 2010), primarily patients with UWS (Dolce et al., 2008), and have not investigated differences between clinical conditions. Secondly, the existing studies investigate HRV responses to a single and prolonged exposure to affective stimuli, which could be influenced by eventual reactions from the patients and fatigue resulting from the lengthy experimentation. Finally, most studies have only employed auditory stimuli (Gutiérrez et al., 2010; Riganello and Candelieri, 2010; Riganello et al., 2010), yet incorporating audiovisual stimuli could provide a multisensory experience that enhances engagement, comprehension, and memory recall.

We hypothesized that the exposure to multiple personalized and non-personalized affective audiovisual stimuli would evidence different emotional responses in healthy subjects, patients in MCS and patients with UWS. Consequently, the objective of this study was to examine the HRV responses to affective personalized videos featuring acquaintances and non-personalized videos featuring strangers in a group of healthy subjects, patients in MCS, and patients with UWS.

## 2 Materials and methods

### 2.1 Participants

A convenience sample of 19 healthy subjects and 22 patients with DOC participated in this study. Healthy volunteers older than 18 years, with no medical history of cardiovascular disease, mental illness, or hearing/visual loss, were recruited from the staff and acquaintances of the research institute where the study took place. Patients with DOC were recruited from the long-term rehabilitation unit for patients with acquired brain injury of a tertiary referral hospital network, from January 2022 to June 2023. Patients were potential candidates to participate in the study if they were over 18 years old and were diagnosed as being in a MCS or with a UWS through repeated assessments with the CRS-R (Annen et al., 2019), a scale with high interrater reliability (*α* = 0.84, *p* < 0.001) and test–retest reliability (*α* = 0.94, *p* < 0.001) (Giacino et al., 2004). Patients were excluded if they were extremely agitated or had a history of disabling auditory or visual impairments, psychiatric disorders, neurodegenerative diseases or brain lesions prior to the brain injury that led to their current clinical condition.

The study was approved by the Ethics Committee of Universitat Politècnica de València (P03250321) and Hospital Clínic Universitari (NEURORHB/NP2101). Written informed consent was obtained before enrollment from both healthy subjects and the legal representatives of patients with DOC.

### 2.2 Instrumentation

Healthy participants and the legal representatives of the participants with DOC were asked to mention an acquaintance of them, such as a relative or a friend, with a strong emotional connection. Acquaintances were then contacted and asked to provide self-recorded videos talking about 8 specific topics related to (a) anecdotes shared with the participants, (b) their passions, (c) dreams and aspirations, (d) positive qualities and strengths, (e) achievements, (f) experiences that relatives would like to relive, (g) desired plans, and a (h) free speech about things that relatives would like to tell the participants. An experimenter gave specific instructions to the acquaintances, guided them through the process, and recorded the videos when necessary. In case that the acquaintances were not capable to provide the videos, they were asked to mention a substitute. Videos were required to be longer than one minute, to be recorded in landscape orientation and to feature a medium shot of the relatives sitting on a chair with a white background. Six out of the 8 videos demanded to the acquaintance were selected for each participant. Selection was done according to the availability of the videos and compliance with the given instructions, in the order described above. Videos were trimmed to 55 s and normalized in terms of luminosity, color, framing and volume by an experimenter.

The videos were displayed using a virtual reality headset, the HTC VIVE Pro Eye (HTC Corporation, New Taipei City, Taiwan), to ensure that the stimuli were always visible to the participants even if they turned their head. The audio of the videos was presented using the headphones of the virtual reality headset. The controller of this device was also used for the healthy controls to provide feedback about the stimuli, as described below.

A four-lead, five-wire electrocardiogram (ECG) recording system with a sampling rate up to 8 kHz, the Shimmer3 ECG (ConsenSys, Brooklyn, NY, EEUU), was used to record the electrical cardiac activity during the experiment. Other responses were recorded in addition to the ECG, including electrical and hemodynamic brain activity and ocular reactions, which were not analyzed in this particular study.

### 2.3 Procedure

The experiment took place in dedicated and quiet rooms, free of distractors and controlled temperature (24° C) and was conducted and supervised by an experimenter. Participants were briefly introduced to the procedure of the study, informing that they would be viewing a series of videos, but no information was provided about their content or protagonists. Healthy participants were then asked to comfortably seat on a chair with armrests and patients with DOC were transferred to an adapted wheelchair. An experimenter fixed the electrodes of the ECG monitor at the right and left arm and the precordial V5, following the manufacturer guidelines, and, finally, the virtual reality headset. The quality of the registered signals was checked, and any technical issue was solved if necessary. Then, the experiment started.

**Figure 1:**
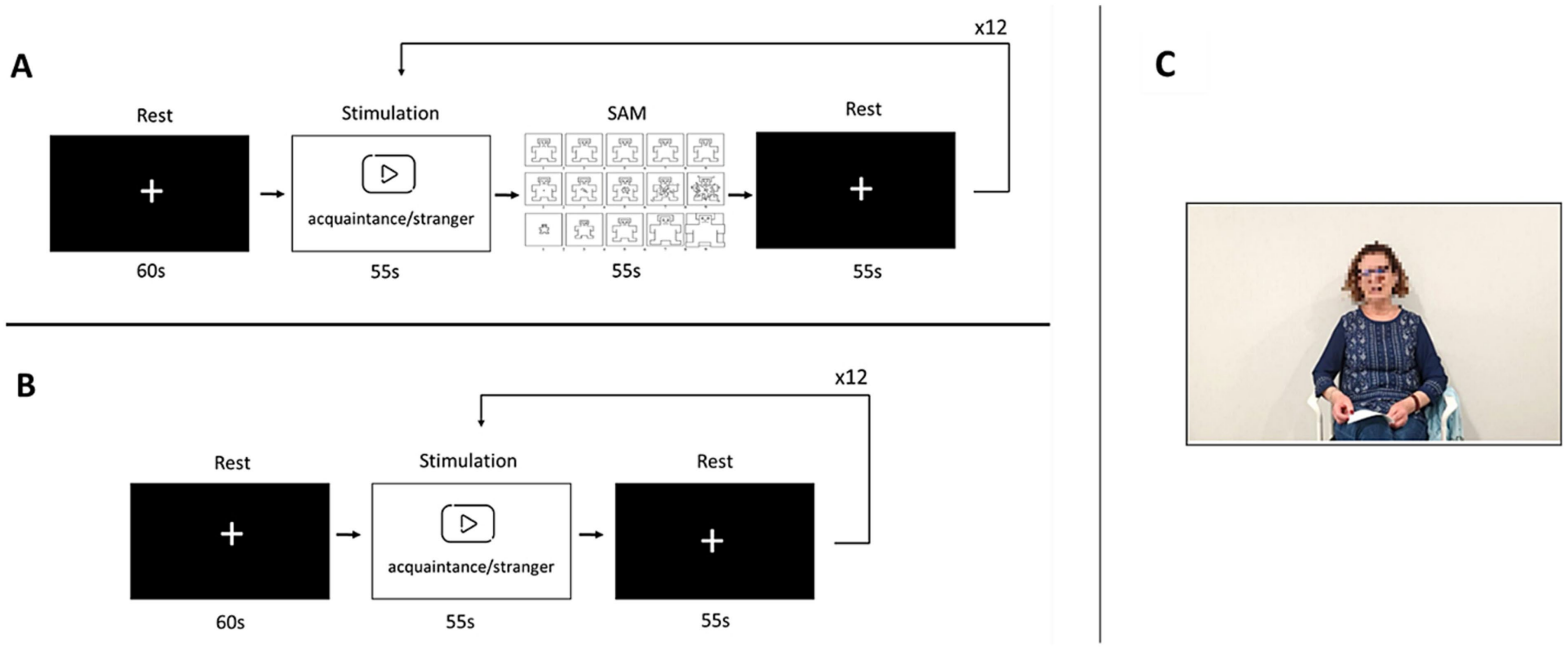
Experimental procedure. Experimental protocol for both healthy controls **(A)** and patients with disorders of consciousness **(B)** involved several steps. First, all participants underwent a 60-s baseline period where they observed a white cross on a black screen while at rest. Subsequently, all participants viewed a series of six 55-s videos. These videos included acquaintances discussing topics relevant to the participants or strangers discussing the same topics relevant to other individuals **(C)**. The order of presentation was randomized. For patients with disorders of consciousness, each video was followed by a 55-s resting period where the same baseline stimulus was displayed. For healthy subjects, each video was followed by a digital version of the SAM scale that participants had to complete within a 55-s time frame and then had a 55-s resting period, consistent with the procedure described earlier. SAM, Self-Assessment Manikin scale.

Before the emotional stimulation, a 60-s baseline period of the ECG activity was recorded while participants were at rest. During this period, a white cross in the center of a black screen was displayed in the virtual reality headset (Figure 1). After this, participants were presented with a series of 12 55-s videos, six of them featuring their acquaintance and other six featuring an unknown person (either an acquaintance of another participant or an actor or actress), who was age and sex-matched. To ensure consistency and minimize potential confounding factors, all videos were normalized in terms of speaker positioning (centred), illumination, and sound levels. This ensured that differences in responses were attributable to the emotional content rather than technical aspects of the videos. This way participants either watched and listened to their acquaintance talking about their characteristics, shared memories and plans, or a stranger talking about another person and unrelated anecdotes. The videos were played in a randomized order to minimize any potential carry-over effects. A 55-s resting period was conducted after each video. During these periods the same white cross over a black screen of the baseline period was displayed in the virtual reality headset.

Figure 1 illustrates the experimental protocol for both healthy controls (A, top left) and patients with disorders of consciousness (B, bottom left). Healthy participants were additionally asked to provide feedback about each video. A virtual version of the Self-Assessment Manikin scale (SAM) (Bradley and Lang, 1994) was implemented and displayed in the virtual reality headset and healthy participants were asked to rate the valence, arousal and dominance of the previous video in three nine-point Likert scales using the controller of the virtual reality headset. Healthy subjects reported that the videos of acquaintances and strangers were different for valence [8.14 ± 0.97 vs. 5.43 ± 1.52; *t* (219) = 17.187; *p* < 0.001], arousal [5.67 ± 2.03 vs. 2.38 ± 1.50; *t* (219) = 13.95; *p* < 0.001] and dominance [4.49 ± 2.07 vs. 6.80 ± 2.47; *t* (219) = −8.94; *p* < 0.001].

If the experimenter considered that the participants fell asleep or detected that they were too agitated, the experiment was temporarily paused. The experimenter tried to restore an adequate level of arousal and agitation and resumed the study if it was achieved or canceled the experiment otherwise.

### 2.4 Data analysis

#### 2.4.1 Signal processing

The ECG recordings of each subject were resampled to 256 Hz. To eliminate low-frequency noise, including respiration-related baseline wander, a 5th order Butterworth high-pass filter with 0.5 Hz cut frequency was applied. Additionally, a 50 Hz notch filter was used to mitigate power line interferences. Then R peaks were detected using the Pan-Tompkins algorithm (Pan and Tompkins, 1985) and were visually inspected for missing beats (Choi and Shin, 2018). Interpolation was used to correct ectopic beats when they accounted for less than 20% of the total beats (Choi and Shin, 2018). To align the inter-beat interval series with the experimental protocol, the data were segmented into 55-s windows, each of them corresponding to the stimuli or resting periods. Ultra-short term HRV measures were further extracted for analysis using the Neurokit2 Python library (Makowski et al., 2021). This process involved computing time-domain and frequency-domain HRV features from short segments of the inter-beat interval series, following the guidelines outlined by (Shaffer et al., 2020). The investigated features are listed in Table 1 along with their descriptions.

**Table 1:** Ultra-short-term heart rate variability features.

	Measure	Description
Time-domain measures	MeanNN (ms)	Mean of the R-R intervals
	MedianNN (ms)	Median of the R-R intervals
	MadNN (ms)	Median absolute deviation of the R-R intervals
	SDNN (ms)	Standard deviation of the R-R intervals
	RMSSD (ms)	Root mean square of successive differences of R-R intervals
	SDSD	Standard deviation of successive differences of R-R intervals
	Prc80NN	80th percentile of the R-R intervals
	Prc20NN	20th percentile of the R-R intervals
	pNN20 (%)	% of successive differences of R-R intervals >20 ms
	pNN50 (%)	% of successive differences of R-R intervals <50 ms
Frequency-domain measures	HF (ms^2^)	Absolute power of the high-frequency band (0.15–0.4 Hz)
	HFn	Normalized HF

List and description of the ultra-short-term heart rate variability features used in the study.

All the HRV measures corresponding to the stimulation periods featuring both acquaintance and strangers were estimated and normalized to the preceding resting period, and then averaged, as described previously (Nardelli et al., 2015). Specifically, for each stimulation period, the value of all the HRV features was computed as the value of each feature in the current stimulation period minus the value of the same features in the preceding resting period. The final value was then divided by the median absolute deviation of the feature for that specific subject.

#### 2.4.2 Statistical analysis

Normality of the data was assessed using Shapiro–Wilk tests, which confirmed non-normal distributions. Wilcoxon tests were conducted to examine differences in HRV measures between videos of acquaintances and strangers, as well as between groups of healthy controls, patients in MCS, patients in UWS, and the combined DOC group (MCS and UWS). Bonferroni corrections were conducted to control for multiple comparisons and reduce the risk of Type I errors. For all statistical comparisons, we reported the medians (Mdn), test statistic values (t or Z), corrected *p*-values, and effect sizes (r). Significant level *α* was set at 0.05. Group-level statistical analyses were not performed due to the unequal sample sizes across groups, which could compromise the validity of such comparisons. Data analysis was conducted using Python, version 3.12.

The limited sample size prevented the application of machine learning for classifying HRV responses, as it would not have yielded robust or generalizable results. Instead, statistical analyses were used to examine group differences and response patterns. The discussion section further addresses the challenges of using machine learning in this context.

## 3 Results

### 3.1 Participants

A total of 19 healthy subjects and 22 patients with DOC participated in the study. The data of two healthy subjects were lost due to technical problems with the ECG recording system. Additionally, the data of 11 patients were discarded from the analysis either because some electrodes were detached by the participants or were extremely affected by noise. As a result, the data of 17 healthy subjects and 11 patients with DOC were included in the analyses. The group of healthy subjects consisted of 9 women and 8 men, with a mean age and standard deviation of 34.65 ± 11.65 years. The group of patients with DOC included seven patients in MCS and six patients with UWS, with a mean age and standard deviation of 35.00 ± 15.81 years. An independent t-test revealed no statistically significant difference in age between the two groups (*t* = −0.05, *p* = 0.96). Table 2 shows the individual demographic and clinical characteristics of this group.

**Table 2:** Demographic and clinical information of patients with DOC.

Patient	Sex	Age (years)	Etiology	Clinical diagnosis	Time since injury (days)	CRS-R
P1	M	35	Anoxia	UWS	817	7
P2	F	32	Anoxia	UWS	152	8
P3	F	31	Anoxia	UWS	281	6
P4	F	19	Anoxia	UWS	126	5
P5	M	18	Anoxia	UWS	676	7
P6	M	57	Anoxia	UWS	903	8
P7	F	73	TBI	MCS	117	11
P8	M	25	TBI	MCS	557	10
P9	M	25	TBI	MCS	2,202	12
P10	F	40	TBI	MCS	8,881	13
P11	M	30	TBI	MCS	210	14

The table describes the sex, age, etiology, clinical diagnosis, days after onset, and baseline CRS-R score of the patients with DOC that participated in the study. F, female; M, male; TBI, traumatic brain injury; UWS, unresponsive wakefulness syndrome; MCS, minimally conscious state.

### 3.2 Differences in the heart rate variability in response to videos of acquaintances and strangers

**Figure 2:**
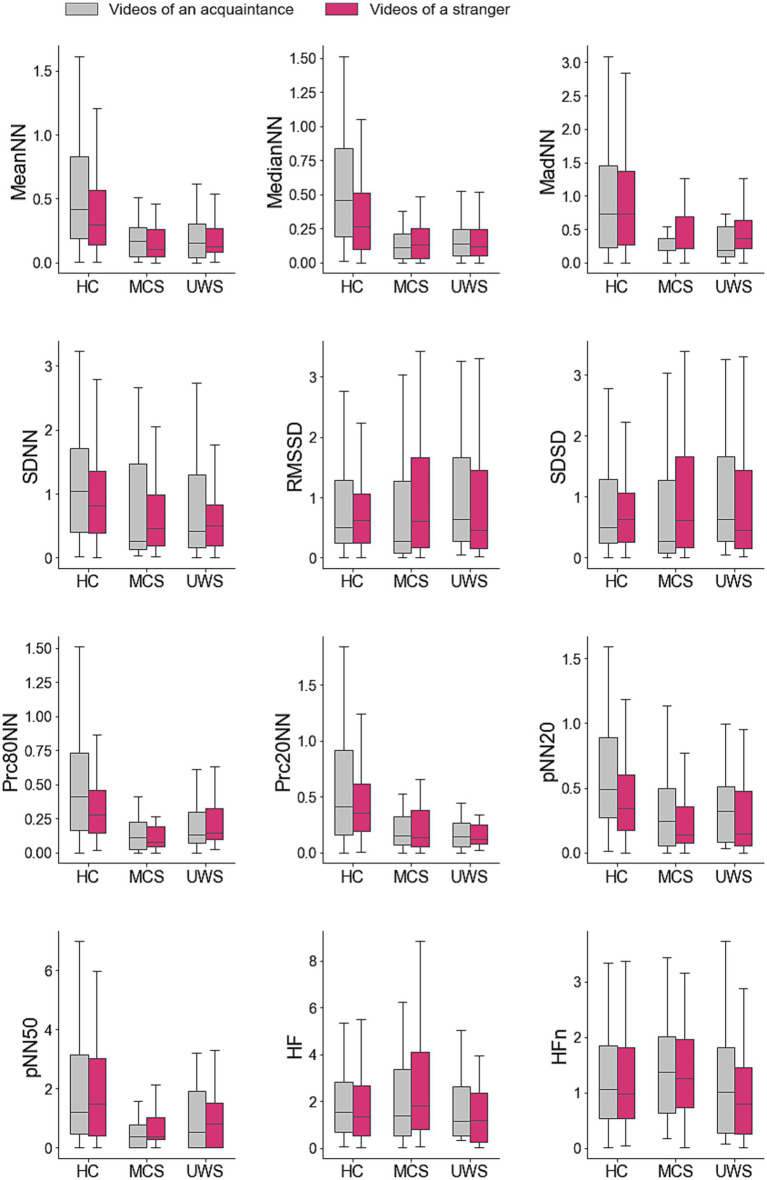
Average values of the heart rate variability measures in response to personalized and non-personalized affective videos. Average difference between the values of the ultra-short term HRV measures registered during the videos and those registered during the previous resting period. Values are provided for groups of participants and type of videos. HC, healthy controls; MCS, patients in minimal consciousness state; UWS, patients with unresponsive wakefulness syndrome.

Table 3 summarizes the test results for comparisons between videos of acquaintances and strangers, showing whether significant differences were found in HRV measures for each group. The average values of the HRV measures associated to videos of acquaintance and strangers of healthy subjects, patients in MCS, and patients with UWS are provided in Figure 2. The responses of healthy subjects to the emotional videos featuring acquaintances and strangers evidenced statistically significant differences in the *meanNN* (Mdn = 0.40 vs. 0.29, Z = 3.98, *p* < 0.001, *r* = 0.17), *medianNN* (Mdn = 0.44 vs. 0.25, Z = 3.89, *p* < 0.001, *r* = 0.22), *pNN20* (Mdn = 0.55 vs. 0.48, Z = 3.53, *p* < 0.001, *r* = 0.06) and *prc80NN* (Mdn = 0.413 vs. 0.243, *Z* = 4.19, *p* < 0.001, *r* = 0.18). However, no differences were found in any HRV measure for patients with DOC.

**Table 3:** Results of HRV measures comparisons across cohorts and stimuli.

HRV measure	Acq vs. Str			HC vs. MCS		HC vs. UWS		HC vs. DOC	
	HC	UWS	MCS	Acq	Str	Acq	Str	Acq	Str
MeanNN (ms)	***	–	–	***	***	***	***	***	***
MedianNN (ms)	***	–	–	***	***	***	***	***	***
MadNN (ms)	–	–	–	***	***	***	***	***	***
SDNN (ms)	–	–	–	***	–	–	–	–	–
RMSSD (ms)	–	–	–	–	–	–	–	–	–
SDSD	–	–	–	–	–	–	–	–	–
Prc80NN	***	–	–	***	***	***	***	***	***
Prc20NN	–	–	–	***	***	***	***	***	***
pNN20 (%)	***	–	–	***	***	***	***	***	***
pNN50 (%)	–	–	–	–	–	–	–	–	–
HF (ms^2^)	–	–	–	–	–	–	–	–	–
HFn	–	–	–	–	–	–	–	–	–

HC, healthy control; UWS, unresponsive wakefulness syndrome; MCS, minimally conscious state; DOC, disorders of consciousness. Acq vs. Str, comparisons between acquaintances and stranger videos. *** indicates significant differences after Bonferroni correction. Comparisons were made for each stimulus and across cohorts.

### 3.3 Sensitivity of the heart rate variability in response to videos of acquaintances and strangers to the clinical condition

Table 3 presents the test results for comparisons between groups in response to videos of both acquaintances and strangers, indicating whether significant differences were observed. Statistical analyses revealed no statistical differences between the HRV responses of patients in MCS and UWS. However, significant differences were found between the group of healthy subjects and the groups of patients in MCS and with UWS in some HRV measures in response to videos of both acquaintances and strangers.

Specifically, significant differences between healthy subjects and patients with MCS were found in the *MeanNN* (videos of acquaintances: Mdn = 0.41 vs. 0.17, Z = 4.32, *p* < 0.001, *r* = 0.39; videos of strangers: Mdn = 0.29 vs. 0.10, Z = 3.58, *p* < 0.001, *r* = 0.32), the *MedianNN* (videos of acquaintances: Mdn = 0.46 vs. 0.11, Z = −4.87, *p* < 0.001, *r* = 0.48; videos of strangers: Mdn = 0.27 vs. 0.13, Z = 3.60, *p* < 0.001, *r* = 0.32), the *MadNN* (videos of acquaintances: Mdn = 0.73 vs. 0.18, Z = 3.50, *p* < 0.001, *r* = 0.32; videos of strangers: Mdn = 0.74 vs. 0.21, Z = 2.83, *p* < 0.001, *r* = 0.25), the *Prc80NN* (videos of acquaintances: Mdn = 0.43 vs. 0.12, Z = 4.73, *p* < 0.001, *r* = 0.43; videos of strangers: Mdn = 0.28 vs. 0.08, Z = 5.18, *p* < 0.001, *r* = 0.458), the *Prc20NN* (videos of acquaintances: Mdn = 0.4147 vs. 0.1563, Z = 3.58, *p* < 0.001, *r* = 0.32; videos of strangers: Mdn = 0.36 vs. 0.14, Z = 3.35, *p* < 0.001, *r* = 0.30), and the *pNN20* (videos of acquaintances: Mdn = 0.49 vs. 0.24, Z = 3.54, *p* < 0.001, *r* = 0.32; videos of strangers: Mdn = 0.34 vs. 0.14, Z = 2.81, *p* < 0.001, *r* = 0.25). An additional difference between these groups of participants was found for the *SDNN* but only in response to videos of acquaintances (Mdn = 1.04 vs. 0.26, Z = 2.67, *p* < 0.001, *r* = 0.24).

Differences between healthy subjects and patients with UWS were also found in the same measures of the HRV response to both categories of videos: the *MeanNN* (acquaintances: Mdn = 0.56 vs. 0.25, Z = 3.99, *p* < 0.001, *r* = 0.34; strangers: Mdn = 0.38 vs. 0.20, Z = 2.89, *p* < 0.001, *r* = 0.25), the *MedianNN* (videos of acquaintances: Mdn = 0.46 vs. 0.14, Z = 4.58, *p* < 0.001, *r* = 0.42; videos of strangers: Mdn = 0.27 vs. 0.12, Z = 2.97, *p* < 0.001, *r* = 0.27), the *MadNN* (videos of acquaintances: Mdn = 0.73 vs. 0.18, Z = 3.41, *p* < 0.001, *r* = 0.31; videos of strangers: Mdn = 0.74 vs. 0.37, Z = 2.72, *p* < 0.001, *r* = 0.25), the *Prc80NN* (videos of acquaintances: Mdn = 0.41 vs. 0.14, Z = 3.40, *p* < 0.001, *r* = 0.31; videos of strangers: Mdn = 0.28 vs. 0.14, Z = 2.56, *p* < 0.001, *r* = 0.23), the *Prc20NN* (videos of acquaintances: Mdn = 0.41 vs. 0.14, Z = 4.00, *p* < 0.001, *r* = 0.37; videos of strangers: Mdn = 0.36 vs. 0.12, Z = 4.30, *p* < 0.001, *r* = 0.39) and the *pNN20* (videos of acquaintances: Mdn = 0.49 vs. 0.33, Z = 2.67, *p* < 0.001, *r* = 0.25; videos of strangers: Mdn = 0.34 vs. 0.15, Z = 2.53, *p* < 0.001, *r* = 0.23).

Differences between healthy controls and patients with DOC in both categories of videos were found in the *MeanNN* (acquaintances: Mdn = 0.42 vs. 0.16, Z = 5.08, *p* < 0.001, *r* = 0.41; strangers: Mdn = 0.29 vs. 0.13, Z = 4.08, *p* < 0.01, *r* = 0.32), the *MedianNN* (acquaintances: Mdn = 0.46 vs. 0.14, Z = 6.27, *p* < 0.001, *r* = 0.51; of strangers: Mdn = 0.27 vs. 0.12, Z = 4.17, *p* < 0.01, *r* = 0.33), the *MadNN* (acquaintances: Mdn = 0.73 vs. 0.18, Z = 4.36, *p* < 0.001, *r* = 0.35; strangers: Mdn = 0.74 vs. 0.32, Z = 3.51, *p* < 0.05, *r* = 0.28), the *Prc80NN* (acquaintances: Mdn = 0.41 vs. 0.12, Z = 5.17, *p* < 0.001, *r* = 0.42; strangers: Mdn = 0.28 vs. 0.12, Z = 4.96, *p* < 0.001, *r* = 0.39), the *Prc20NN* (acquaintances: Mdn = 0.41 vs. 0.14, Z = 4.77, *p* < 0.001, *r* = 0.39; strangers: Mdn = 0.36 vs. 0.13, Z = 4.80, *p* < 0.001, *r* = 0.38), and the *pNN20* (acquaintances: Mdn = 0.49 vs. 0.25, Z = 3.94, *p* < 0.01, *r* = 0.32; strangers: Mdn = 0.34 vs. 0.14, Z = 3.39, *p* < 0.05, *r* = 0.27).

## 4 Discussion

This study examined and compared the HRV responses of a group of healthy subjects, patients in MCS, and patients with UWS to personalized videos featuring acquaintances and non-personalized videos featuring strangers. Significant differences emerged between the responses of healthy subjects to both stimuli, but not for patients with DOC. The comparison of the responses of the three groups of participants to the personalized and also to the non-personalized stimuli evidenced significant differences between the responses of healthy subjects and those of patients in MCS and with UWS in some HRV measures.

### 4.1 Differences in the heart rate variability in response to videos of acquaintances and strangers

The higher *meanNN* and *medianNN* values observed in the HRV responses of healthy subjects when watching affective videos of acquaintances compared to those of strangers align with prior research exploring variations in HRV features during exposure to emotional stimuli (Shi et al., 2017; Zhu et al., 2019). This observation is consistent with the well-established link between heart rate and emotion regulation (Kreibig, 2010).

Although the *Prc80NN* and *pNN20* have not been investigated in previous research, these features were also found to be higher for personalized stimuli than for non-personalized stimuli in the present study. The *Prc80NN* offers valuable insights into the distribution of heart rate intervals, enabling a thorough comprehension of the variability in heart rate patterns and the assessment of autonomic nervous system dynamics (Shaffer and Ginsberg, 2017). The *pNN20* is closely linked to the activity of the parasympathetic nervous system (Baek et al., 2015). Lower *pNN20* values typically indicate heightened parasympathetic activity and increased sympathetic dominance (Shaffer and Ginsberg, 2017), which highlights the relevance of this feature in the autonomic regulation. Interestingly, the *pNN20* has been shown to be correlated with the *RMSSD* and the *HF power* (Shaffer and Ginsberg, 2017) and be equivalent to the pNN50 (Hutchinson, 2003), although these features did not show differences between stimuli in our study. Several studies, however, have investigated and reported findings related to *pNN5*0. Specifically, the *pNN50* have been shown to vary in response to joy and fear (Valderas et al., 2015), while increasing with fear and anger, and decreasing with happiness (Zhu et al., 2019).

Prior research has applied machine learning algorithms to classify emotions based on HRV responses (Hasnul et al., 2021). Most studies utilized stimuli with contrasting valences, such as negative and positive stimuli, or varying arousal levels (Katsigiannis and Ramzan, 2017; Miranda-Correa et al., 2018; Subramanian et al., 2016) potentially leading to more distinct responses that are hypothetically easier to discern. Secondly, earlier studies employed longer stimuli, spanning several tens of minutes (Katsigiannis and Ramzan, 2017; Miranda-Correa et al., 2018; Subramanian et al., 2016). These longer stimuli offer larger datasets for analysis, which can enhance statistical stability, improve the detection of emotions unfolding over time, and provide better resilience to noise or individual variability. Certain studies integrate HRV features with other peripheral and central responses, which can offer additional and complementary information, thereby enhancing the classification performance (Ali et al., 2018; Goshvarpour et al., 2017; Jang et al., 2015; Lee and Yoo, 2020; Pinto et al., 2020). Lastly, however, it should be taken into account that some studies failed to report or assess the classification performance on data not included in the training set, which could result in higher accuracy but less reliability. The limited sample size and the choice of stimuli in our study, designed to respect ethical considerations and prevent fatigue among patients with DOC, discouraged the use of machine learning techniques due to the risk of overfitting.

The lack of statistical distinctions between responses to personalized and non-personalized stimuli in patients with DOC differed from what was observed in healthy subjects. The limited research on patients with DOC, which typically involves lengthy stimuli with singular repetitions, has consistently focused on the *nuLF* (Dolce et al., 2008; Gutiérrez et al., 2010; Riganello and Candelieri, 2010). However, the duration of the audio stimuli used in our study, which was half the recommended duration for estimating this feature (Shaffer and Ginsberg, 2017), hindered the investigation of this and other short-term measures.

Furthermore, as mentioned, certain studies have shown significant differences in HRV measures when patients were exposed to music with varying levels of arousal and valence. For instance, the intensity of the *nuLF* values has been found to be correlated with the quality of emotional responses, whether positive or negative, to classical music (Riganello and Candelieri, 2010). Some studies (Dolce et al., 2008; Riganello and Candelieri, 2010) have applied machine learning classifiers to differentiate *nuLF* responses to emotional stimuli in patients with UWS. However, these studies faced challenges, as they often relied on only one or two samples per subject for each stimulus type, resulting in classification accuracies that were not high enough to draw firm conclusions. Additionally, an increase in SDNN and RMSSD has been also reported after two weeks of music stimulation (Lee et al., 2011).

### 4.2 Sensitivity of the heart rate variability in response to videos of acquaintances and strangers to the clinical condition

The differences observed between several HRV measures in the responses of healthy subjects to both personalized and non-personalized videos compared to those of patients in MCS and patients with UWS contrast with the absence of differences between the responses of both groups of patients. This finding suggests that the emotional processing of healthy subjects, regardless of whether they are exposed to personalized or non-personalized videos, may differ from that of patients. In simpler terms, this discovery evidences that disorders of consciousness do affect the HRV responses to affective stimuli. This finding is supported by previous studies that showed differences in HRV responses to emotional stimuli between healthy subjects and patients with DOC (Riganello et al., 2010; Riganello and Candelieri, 2010).

### 4.3 Constraints on generality

The findings of this study must be interpreted with caution considering its previously mentioned limitations. Firstly, although the sample size is comparable or even greater than that in other studies (Dolce et al., 2008; Gutiérrez et al., 2010; Riganello et al., 2015) it can still be considered limited, especially when considering individual patient groups. The movements and agitation of the patients, particularly among those in MCS, challenge the accurate recording of biosignals using wearable or attached sensors. This complication, together with the limited prevalence and access to patients with DOC, severely restrict the availability of large datasets for analysis. As mentioned, the data available in our study, consequently, prevented from using machine learning techniques, as their results could be affected by overfitting. Secondly, the exclusive use of positively-valenced videos in our study, either featuring acquaintances or strangers, may have limited the HRV variability. Using stimuli with varying valences and arousal levels might have enhanced the ability to distinguish HRV responses across different stimuli. However, the ethical implications of their use in patients with DOC, who cannot express their rejection to participate or their desire to stop the experimentation, should be considered (Steinert and Friedrich, 2020; Young et al., 2022). Thirdly, the use of short-duration stimuli restricted the data available for analysis, potentially increasing susceptibility to noise and other events, and limited investigation into ultra-short-term HRV responses. However, longer stimuli could exacerbate patient fatigue and distractibility, which can especially detrimental in patients with DOC, who can have severe attention deficits. Additionally, although patients were on a stable medication regimen during the experiments, we could not control for potential confounding factors such as sleep disturbances, medication effects on the autonomic nervous system and HRV, or other physiological alterations inherent to the DOC population. Lastly, while this study focused on HRV measures, exploring concurrent peripheral and central responses such as electrical and hemodynamic brain activity and ocular reactions could offer a more comprehensive understanding of patient responsiveness, a direction for future research. Despite these limitations, our results suggest that healthy subjects tend to exhibit more pronounced HRV responses to emotional stimuli compared to individuals with DOC.

## 5 Conclusion

This study analyzed and compared the HRV responses of healthy subjects, patients in MCS, and patients with UWS to personalized videos of acquaintances and non-personalized videos of strangers. Our results revealed distinct responses to both stimuli in healthy subjects. Additionally, the responses of healthy subjects to each specific stimulus differed from those of patients in MCS and those with UWS. These findings suggest impaired emotional processing in patients with DOC, warranting further investigation in future studies to improve diagnostic approaches for this population.

## Data Availability

The datasets presented in this study can be found in online repositories. The names of the repository/repositories and accession number(s) can be found at: https://osf.io/fqbtr/.
